# The diagnosis and management of solid pseudopapillary epithelial neoplasm of the pancreas in a resource-limited setting: two cases from Cameroon

**DOI:** 10.1093/jscr/rjae032

**Published:** 2024-02-21

**Authors:** Chinonso P Shu, George F F Ngock, Masango M G Lisongwe, Nkwayeb R Ndayong, Arnaud R Djomaleu, Macky F E Yecke, James A Brown

**Affiliations:** Pan-African Academy of Christian Surgeons, Mbingo Baptist Hospital, Bamenda, Cameroon; Pan-African Academy of Christian Surgeons, Mbingo Baptist Hospital, Bamenda, Cameroon; Department of Surgery, Mbingo Baptist Hospital, Bamenda, Cameroon; Pan-African Academy of Christian Surgeons, Mbingo Baptist Hospital, Bamenda, Cameroon; Department of Surgery, Mbingo Baptist Hospital, Bamenda, Cameroon; Department of Clinical Services, Cameroon Baptist Convention Health Services, Bamenda, Cameroon; Department of Clinical Services, Cameroon Baptist Convention Health Services, Bamenda, Cameroon; Pan-African Academy of Christian Surgeons, Mbingo Baptist Hospital, Bamenda, Cameroon; Department of Surgery, Mbingo Baptist Hospital, Bamenda, Cameroon

**Keywords:** solid pseudopapillary epithelial neoplasm of the pancreas, pancreaticoduodenectomy, splenic vein thrombosis, immunohistochemistry, telepathology

## Abstract

Solid pseudopapillary epithelial neoplasm (SPEN) of the pancreas is a rare tumor of low malignant potential that occurs most often in young females. Imaging and histopathology are necessary to confirm the diagnosis as most have no symptoms. Lack of access to these technologies in sub-Saharan Africa contributes to the difficulty in making an early and accurate diagnosis, and hence, impedes treatment. We present two cases of SPEN of the pancreas in young female patients at a rural, teaching hospital in Cameroon. The diagnosis was made only with histopathology. Computed tomography scan with intravenous contrast was essential to planning a safe surgical resection. Both patients had complete surgical resection with good results.

## Introduction

Solid pseudopapillary epithelial neoplasm (SPEN) of the pancreas usually occurs in the tail of the pancreas of young women in the second and third decades of life [1–4]. This tumor was first described in 1959 by Frantz [5] and makes up 1%–2% of pancreatic exocrine tumors [2, 6]. These tumors are slow-growing and usually remain asymptomatic until they are very large. The diagnosis is rarely made until they cause pain and abdominal fullness or mass. They are associated with cystic degenerative changes at the time of diagnosis [3, 7]. Computed tomography (CT) scan may be nonspecific in up to 40% of cases [6]. Immunohistochemistry is necessary for a definitive diagnosis [8]. This is important as liver enzymes and tumor markers are usually normal [4, 6]. The liver is the most common site of metastases which occurs in an infiltrative pattern in about 15% of cases [1, 6]. Surgery is the mainstay of management even in the presence of metastases [1, 3, 6].

## Case reports

**Figure 1:**
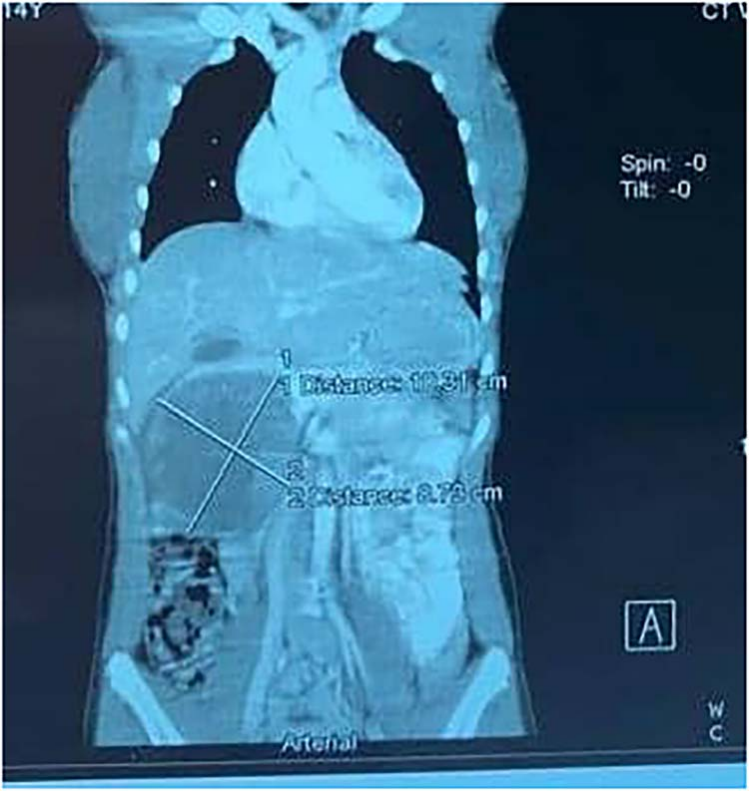
A contrast-enhanced CT scans of the abdomen showing a large, well-defined, and heterogeneous tumor.

A 14-year-old female with no relevant past medical history presented with a 2-week history of right upper quadrant abdominal swelling. She gave no history of trauma, pain, fever, jaundice, alteration in bowel habits, early satiety, bloating, or anorexia. On physical examination, she appeared healthy. Her vital signs were normal, and she was anicteric. The rest of her exam was normal except for a right upper quadrant fullness and a palpable liver edge 6 cm below the right costal margin. An abdominal ultrasound was interpreted as a right lobe liver mass. A fine needle aspirate revealed necrotic tissue with no hepatocytes seen. A thoraco-abdominal CT scan revealed an enhancing, centrally necrotic retroperitoneal mass of 9 × 12 cm compressing the duodenum, portal vein, and inferior vena cava. Mild adjacent free fluid was noted. There was no evidence of metastatic disease (Fig. 1). All routine blood tests including complete blood count, chemistries (amylase and lipase inclusive) and coagulation studies were normal. A gastrointestinal stromal tumor was suspected.

**Figure 2:**
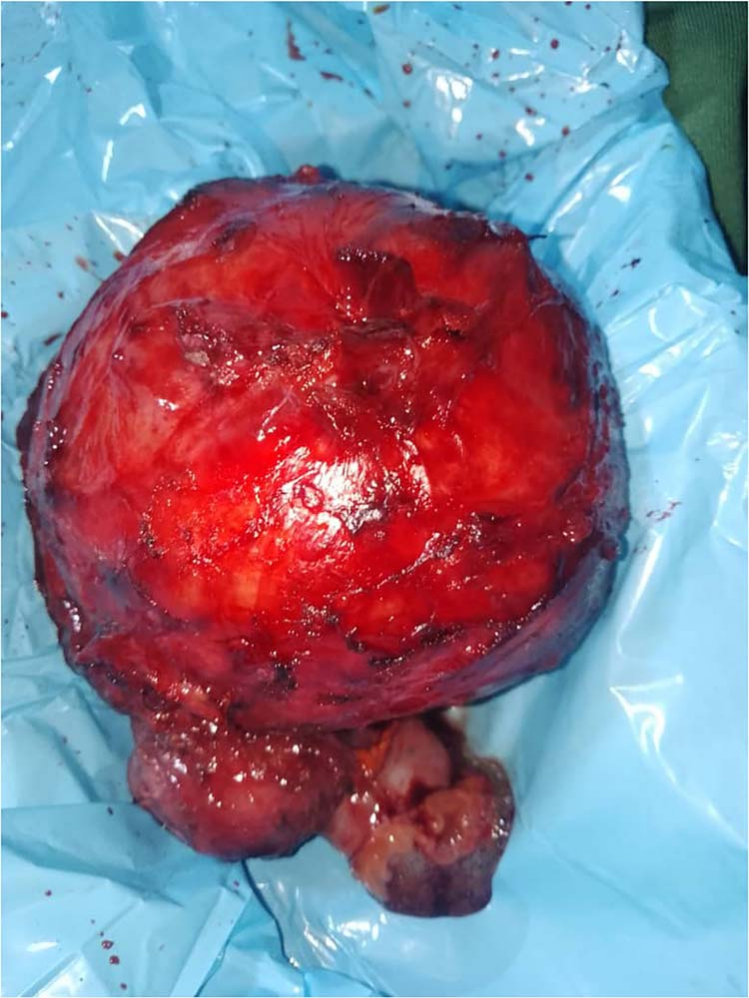
Anatomical piece showing a well-defined resected tumor.

At surgery, she was found to have a large sub-hepatic mass extending into the lesser sac and arising from the head of the pancreas. The contents were necrotic and hemorrhagic. An incisional biopsy was taken along with several peri-hepatic lymph nodes. Histopathology returned with a diagnosis of SPEN of the pancreas. All four lymph nodes were negative for metastatic tumor. At a second laparotomy, a pancreaticoduodenectomy (Whipple procedure) was performed (Fig. 2). Her postoperative course was complicated by a pancreatic leak that resolved on postoperative Day 5, and a superficial postoperative wound infection. She was discharged on postoperative Day 15. Histopathology revealed negative margins and lymph nodes, and she was euglycemic.

**Figure 3:**
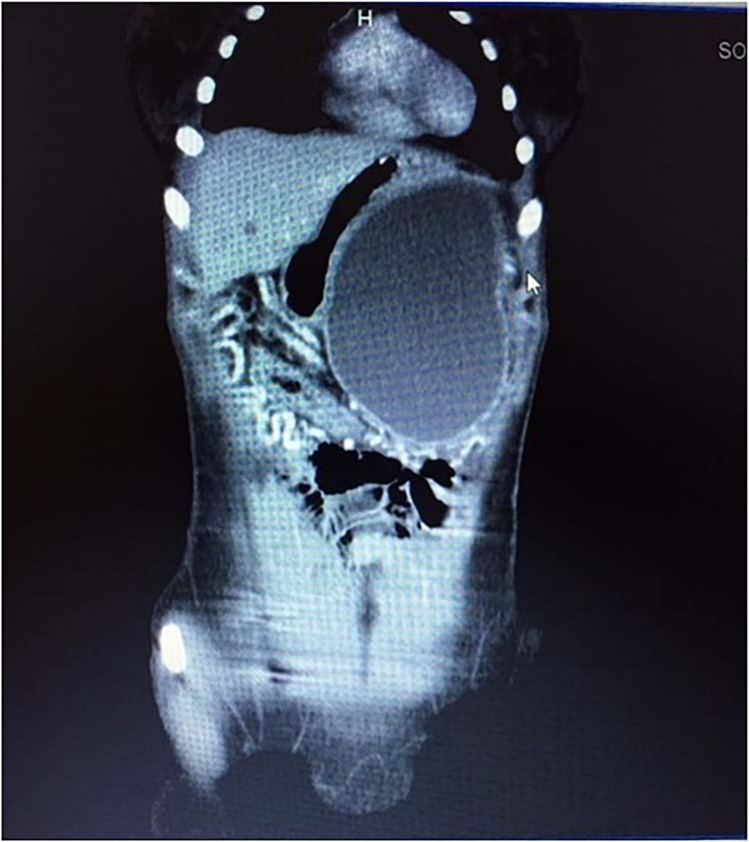
CT scan showing mass with well-defined borders.

**Figure 4:**
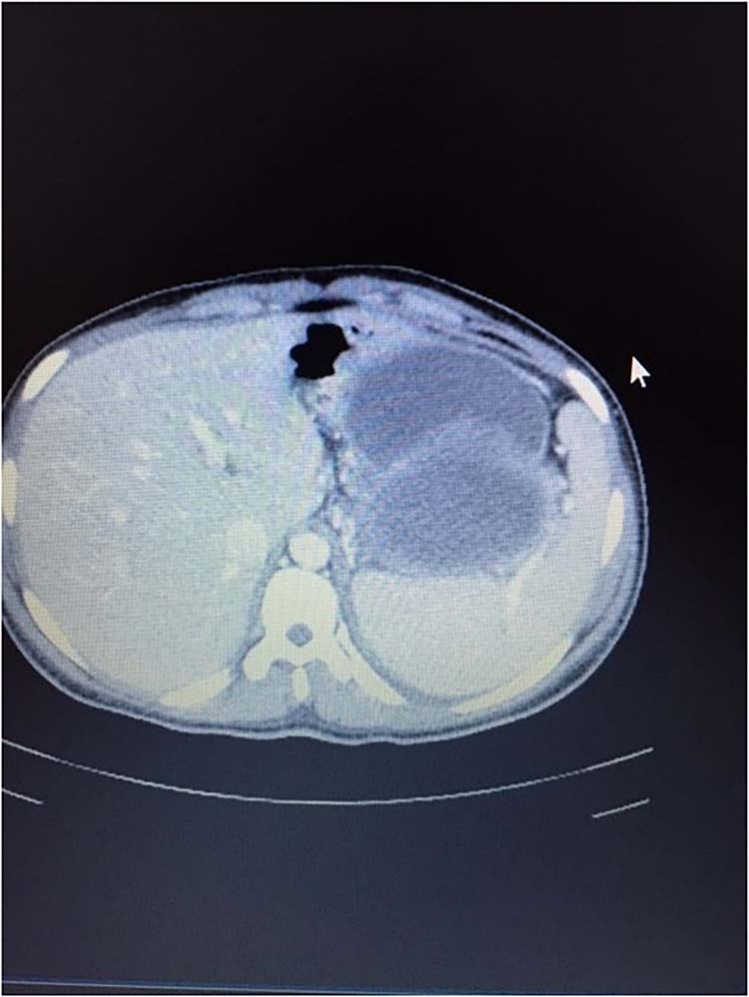
CT scan showing normal liver and enlarged spleen in close proximity with the mass.

A 21-year-old female collapsed in a market and was taken to a local hospital where she was told that she had a ruptured spleen. She was transfused with two units of whole blood and discharged without surgery. Four weeks later, she noted persistent fullness in her left upper quadrant and presented to our hospital for further evaluation. A contrast CT scan revealed two well-encapsulated, cystic masses in the left upper quadrant, the largest one with small solid components abutting the tail of the pancreas and the smaller one adjacent to the posterior wall of the stomach. She had splenic vein thrombosis, but her portal vein was patent. Numerous portosystemic venous pathways were present, mostly in the gastrosplenic area. The spleen was homogeneous but enlarged with a smooth capsule and normal contour. The liver was normal (Figs. 3 and 4). Her preoperative laboratory studies, including complete blood count, liver function tests, and coagulation studies were normal. A fine-needle aspiration of the anterior mass revealed fresh blood. She was given vaccines against pneumococcus, meningococcus, and haemophilus, anticipating splenectomy would be necessary to resect this mass. At surgery, the splenic artery was ligated at its origin from the celiac axis, which led to an immediate decompression of the peri-splenic and gastric venous collaterals before attempting resection of the mass. The anterior/superior mass was a large, partially organized hematoma, but the inferior mass arose from the tail of the pancreas. A distal pancreatectomy and splenectomy were done en-bloc. The final histopathology revealed a solid pseudopapillary epithelial tumor of the pancreas with negative margins of resection. The patient had an uncomplicated postoperative course and remains asymptomatic.

## Discussion

Solid pseudopapillary epithelial tumors of the pancreas are rare and occur most often in young women, as our two cases demonstrate [4]. Most of these tumors arise in the tail of the pancreas but can arise in the head of the pancreas, as our first case demonstrates. Hepatobiliary obstruction is rare but can occur by extrinsic compression from large tumors. The most common presentation is abdominal pain and fullness [7, 9]. Preoperative diagnosis is difficult, and CT and magnetic resonance imaging scans can be mis-interpreted in up to 40%–50% of cases [6, 9]. These tumors are often mistaken for gastrointestinal stromal tumors preoperatively, which highlights the need for histopathology and immunohistochemical markers which are not readily available in sub-Saharan Africa [10]. Telepathology has helped to breach the gap in such settings [11], which is true for our hospital with one pathologist who is sometimes away. Tissue for histopathology can be obtained either preoperatively or after excision, as in our case; as some authors argue preoperative tissue diagnosis could lead to extra-pancreatic spread of the disease despite the fact that another school of thought favors this as it helps plan the surgery and prevents going in twice as in our case [12, 13]. Histopathology also helps to differentiate benign and malignant forms of the disease aided by immunohistochemical markers of which vimentin, alpha-1-antitrypsin, neuron specific enolase(NSE), CD10 and CD56 are particularly helpful [8, 12]. Malignant features include vascular and nerve sheath infiltration, lymph node metastases, and liver metastases [14]. There is still controversy over the clinical predictability and prognosis of malignant pathologic findings in these patients. [15]. Complete surgical excision is the mainstay for cure with the operation tailored to the size and location of the primary tumor [6]. Our first patient required pancreaticoduodenectomy which is rare in teenagers [16]. Neoadjuvant and adjuvant chemotherapy have been beneficial in some cases while chemoradiation has been used in unresectable cases [6]. The prognosis is favorable even in the presence of distant metastases. Recurrence rates are low, and 5 year survival as high as 97% [6, 17].

## Conclusion

SPEN of the pancreas are rare, slow-growing tumors that usually occur in young women. Contrast imaging and histopathology, especially immunohistochemical staining, are essential to the diagnosis. Lack of access to these modalities in sub-Saharan Africa complicates the diagnosis and management of these tumors. Surgical resection with an R0 excision is the mainstay of treatment and results in an excellent long-term prognosis.

## Data Availability

This is a case study so all data used are within the article.
